# Gestational Ectopic Hyperparathyroidism: A Case Report of Perioperative and Perinatal Outcomes

**DOI:** 10.7759/cureus.56406

**Published:** 2024-03-18

**Authors:** Kimitaka Shibue, Nozomi Kubo, Hiromi Oda, Etsuko Tanabe, Tsutomu Okada, Cheng-long Huang, Toshihiro Higuchi, Akihiro Hamasaki, Nobuya Inagaki

**Affiliations:** 1 Department of Endocrinology and Diabetes, Medical Research Institute Kitano Hospital, PIIF Tazuke-Kofukai, Osaka, JPN; 2 Department of Obstetrics and Gynecology, Medical Research Institute Kitano Hospital, PIIF Tazuke-Kofukai, Osaka, JPN; 3 Department of Thoracic Surgery, Medical Research Institute Kitano Hospital, PIIF Tazuke-Kofukai, Osaka, JPN; 4 Department of Radiology, Kansai Electric Power Hospital, Osaka, JPN

**Keywords:** ectopic parathyroid tissue, hyperparathyroid, pregnancy and hypercalcemia, surgery in pregnancy, parathyroid gland adenoma

## Abstract

In the realm of obstetric care, discerning the subtle signs of primary hyperparathyroidism (PHPT) amidst common pregnancy symptoms remains a formidable challenge. Our exploration into a case of gestational hypercalcemia peels back the layers of this complexity, revealing the clinical conundrum posed by overlapping gastrointestinal manifestations. The journey from diagnosis through surgical intervention to the resolution of symptoms underscores the importance of vigilance for PHPT in pregnant patients. This case further prompts consideration of gamma-aminobutyric acid (GABA) as a potential piece in the puzzle of persistent symptoms post-calcium normalization, inviting a broader dialogue on the intricacies of parathyroid pathology in pregnancy.

## Introduction

Primary hyperparathyroidism (PHPT) is a prevalent endocrine disorder, where the parathyroid hormone (PTH) is secreted autonomously because of adenoma or hyperplasia in the parathyroid gland [1,2]. PHPT is characterized by hypercalcemia and elevated or inappropriately normal serum levels of PTH. In the majority of cases, approximately 80%, the condition is attributed to a single parathyroid adenoma. Hyperplasia involving all four glands is responsible for about 10-15% of cases [2].

There is a gender and racial bias, with a higher incidence in females and African Americans [3]. Although most cases of PHPT are nonhereditary and of unknown etiology, some cases have genetic pathogenesis [4,5], especially when presenting as part of multiple endocrine neoplasia type 1 (MEN1) [6]. Therefore, a careful systemic investigation is required for the early detection of any coexisting endocrinological disorders. Although PHPT can occur in patients with diverse backgrounds, gestational hyperparathyroidism is a rare complication that can arise during pregnancy [7]. Pregnancy can lead to a decrease in total calcium concentrations due to the increase in plasma volume and the subsequent reduction in albumin levels. However, the levels of ionized (free) and albumin-adjusted calcium are not affected in pregnant females with normal parathyroid function, and these values can be used for making clinical decisions related to calcium and parathyroid-related disorders during pregnancy [8]. Of note, the symptoms related to hypercalcemia due to hyperparathyroidism can sometimes present with symptoms that overlap with typical gastrointestinal symptoms experienced during pregnancy, such as nausea and vomiting. This may result in a possible postponement in identifying and treating this significant condition in pregnant females.

Gestational primary hyperparathyroidism is reported to be present in 0.15-1.4% of pregnancies [9]. Despite the critical importance of early intervention for gestational primary hyperparathyroidism, the rarity of the condition and challenges in early diagnosis - due to overlapping physical symptoms - limit the available evidence on managing hyperparathyroidism during pregnancy. Most of this evidence comes from observational studies.

To treat maternal hypercalcemia, a regimen of intravenous fluids and specific diuretics is effective for increasing calcium excretion. Although calcitonin and bisphosphonates have been used, their application during pregnancy is limited due to potential risks, including transient benefits and concerns about fetal bone development [10].

In this study, we underscore the imperative management of primary hyperparathyroidism during pregnancy, demonstrated through a case study of a 39-year-old patient. The successful resolution of gestational PHPT in this case underscores the necessity for prompt diagnosis and effective treatment strategies to mitigate potential maternal and fetal complications. Furthermore, this study introduces a novel observation - the correlation between serum calcium/parathyroid hormone levels and gamma-aminobutyric acid (GABA) fluctuations.

## Case presentation

A 39-year-old female, six weeks pregnant, was admitted to our hospital with persistent symptoms of nausea, vomiting, and loss of appetite over the past week. The symptoms began acutely and were not accompanied by abdominal or chest pain, melena, or hematemesis.

Her medical history was notable for urinary calculus in her teenage years and hemolysis, elevated liver enzymes, and low platelets (HELLP) syndrome during her first delivery at 36 years of age. She had no familial history of endocrinological disorders and reported never smoking or drinking alcohol. Additionally, she was not on any regular medication, including over-the-counter drugs.

Upon admission, examinations revealed a significant weight loss, the presence of ketones in her urine, hypercalcemia, and a low phosphorus level. An endocrinological evaluation indicated elevated levels of intact PTH and 1.25-(OH)_2_ vitamin D (Table 1).

**Table 1:** Laboratory test results on admission and post-operative day 1 (POD1) following parathyroidectomy are presented. Px: parathyroidectomy; HCT: hematocrit; PLT: platelets; TP: total protein; Alb: albumin; T-bil: total bilirubin; AST: aspartate aminotransferase; ALT: alanine aminotransferase; LD: lactate dehydrogenase; CK: creatine kinase; Na: sodium; K: potassium; Cl: chloride; Ca: calcium; P: phosphorus; BUN: blood urea nitrogen; Cre: creatinine; eGFR: estimated glomerular filtration rate; CRP: C-reactive protein; Glu: glucose; i-PTH: intact parathyroid hormone; PTHrP: parathyroid hormone-related protein; FT4: free thyroxine; TSH: thyroid-stimulating hormone; 1.25-(OH)2VitD: 1.25-dihydroxyvitamin D

Variables		Admission	POD1 Px	Normal range
CBC	WBC	6400/μL	7800/μL	4000-9000/μL
	RBC	4740000/μL	2840000/μL	3770000-5550000/μL
	Hb	14.5 g/dL	8.7 g/dL	12-16 g/dL
	HCT	43.8%	26.1%	35-45%
	PLT	184000/μL	114000/μL	150000-450000/μL
Biochemical tests	TP	6.2 g/dL	4.5 g/dL	6.5-8.0 g/dL
	Alb	3.9 g/dL	2.3 g/dL	>4.0 g/dL
	T-Bil	1.3 mg/dL	0.7 mg/dL	0.4-1.5 mg/dL
	AST	14 U/L	12 U/L	7-38 U/L
	ALT	10 U/L	6 U/L	4-44 U/L
	LD	102 U/L	149 U/L	120-245 U/L
	CK	27 U/L	167 U/L	41-153 U/L
	Na	138 mEq/L	136 mEq/L	137-147 mEq/L
	K	4.6 mEq/L	3.6 mEq/L	3.5-5.0 mEq/L
	Cl	109 mEq/L	108 mEq/L	98-108 mEq/L
	Ca	13.4 mEq/L	9.2 mEq/L	8.4-10.4 mEq/L
	P	2 mEq/L	2.5 mEq/L	2.5-4.5 mEq/L
	BUN	21.9 mEq/L	31 mEq/L	8-20 mEq/L
	Cre	0.54 mEq/L	0.37 mEq/L	0.4-0.8 mEq/L
	eGFR	98 mL/min/1.73 m^2^	149 mL/min/1.73 m^2^	60 mL/min/1.73 m^2^
	CRP	0.03 mg/dL	3.07 mg/dL	<0.5 mg/dL
	Glu	81 mEq/L	88 mEq/L	70-110 mEq/L
	HbA1c	4.9%	N/A	<6.2%
Endocrinological tests	i-PTH	183 pg/mL	25.5 pg/mL	10-65 pg/mL
	PTHrP	<1.0 pmol/L	N/A	<1.1 pmol/L
	FT4	1.06 ng/dL	N/A	0.90-1.70 ng/dL
	TSH	0.343 µLU/mL	N/A	0.50-5.00 µLU/mL
	1.25-(OH)_2_VitD	124 pg/mL	N/A	20.0-60.0 pg/mL

The 24 h urinary calcium and creatinine levels suggested hypercalciuria, consistent with PHPT. Based on these findings, a diagnosis of PHPT was established. Since PHPT during pregnancy is considered to increase the risk of miscarriage, parathyroidectomy has been reported as a treatment option during pregnancy [10,11]. Thus, we decided to surgically remove the parathyroid gland which was responsible for hyperparathyroidism.

**Figure 1:**
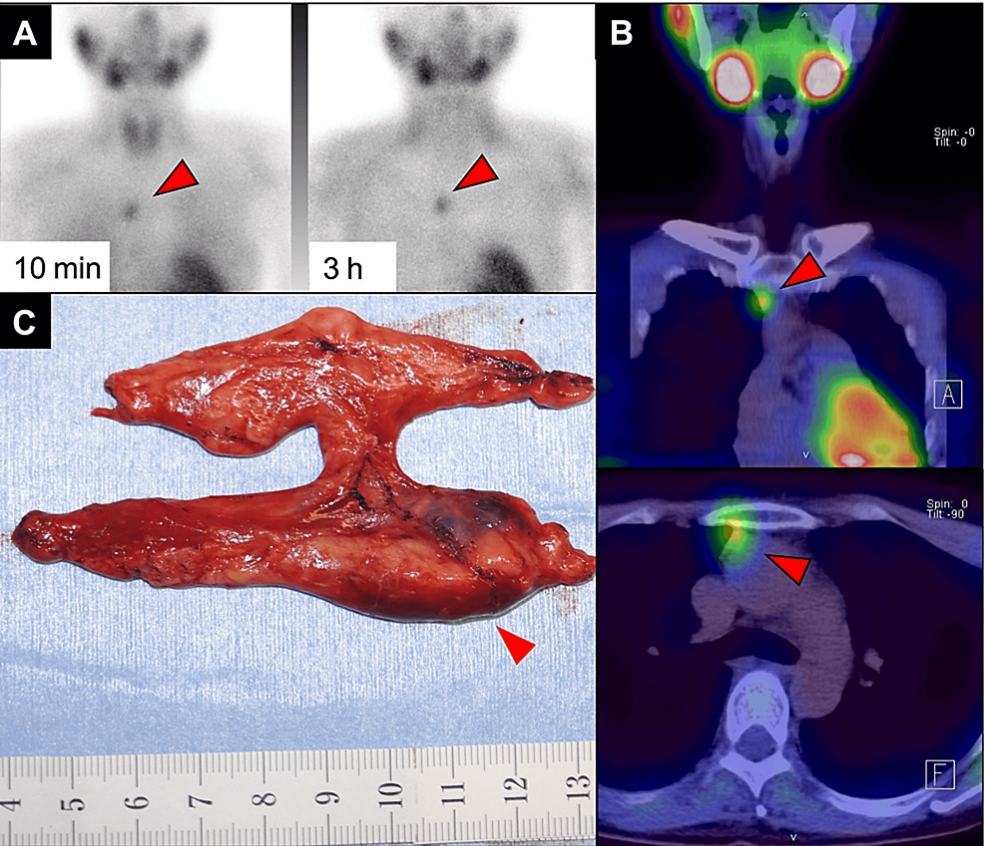
Diagnostic and surgical visualization of parathyroid adenoma with 99mTc-MIBI scintigraphy, SPECT/CT, and gross pathology. Slices of technetium-99m-labeled methoxy isobutyl isonitrile (99mTc-MIBI) scintigraphy. A Symbia T6 (Erlangen, Germany: Siemens Healthineers) single-photon emission computed tomography/computed tomography (SPECT/CT) system was used. (A) A whole-body scan of anteroposterior (AP) view is shown. An early phase of 10 min after injection (left) and delayed phase of 3 h after injection (right) were shown. The focus of intense activity in the right anterior mediastinum (arrowhead) corresponds to the parathyroid adenoma of the patient described in the text. (B) Delayed phase SPECT/CT fusion image. The spot in the mediastinum (arrowhead) corresponds to the parathyroid adenoma of the patient described in the text. (C) Gross image of the extracted thymus containing the nodule of parathyroid adenoma (arrowhead). The thymus and nodule were excised en bloc.

In preoperative examinations, we prioritized the safety of pregnancy and avoided invasive tests as much as possible. To identify the site of the parathyroid adenoma, we performed cervical CT with radiation shielding below the neck. However, we could not identify any hyperplastic parathyroid glands (image not shown). Subsequently, we conducted scintigraphy with technetium-99m-labeled methoxy isobutyl isonitrile (99mTc-MIBI) at 13 weeks and two days, with a safe radiation dose following a discussion with radiologists. The patient received an intravenous injection of 600MBq 99mTc-MIBI. According to the International Commission on Radiological Protection (ICRP) recommendation, estimated whole-body radiation exposure dose of the patient from 99mTc-MIBI and additional CT was considered to be far below the teratogenesis threshold during pregnancy. The scintigraphy demonstrated a hyperintense imaging focus in the anterior mediastinum, indicating an ectopic parathyroid adenoma (Figures 1A-1C).

At 16 weeks of pregnancy, the patient underwent surgical resection of the parathyroid adenoma. An intraoperative rapid blood test directly after the resection showed a rapid decrease of intact PTH in comparison with the previous day. The serum calcium level did not show a rapid decrease in the same test but normalized one day after surgery (Table 1). We used short-term alfacalcidol and calcium lactate hydrate after surgery for the prevention of post-operative hypocalcemia. Both medications were discontinued after confirming a normalized calcium level.

**Figure 2:**
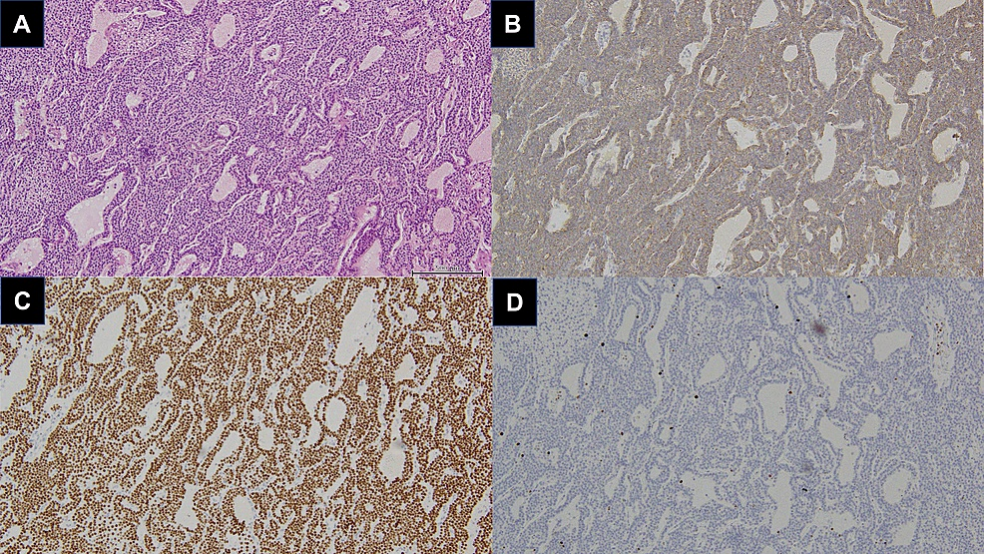
Histological images of the resected ectopic parathyroid adenoma. (A) Hematoxylin and eosin (H&E) staining, (B) chromogranin A staining, (C) GATA3 staining, and (D) MIB-1 staining are shown. A scale bar is included in the bottom-right of image (A) for size reference.

Regarding pathological examination, the resected specimen exhibited alveolar proliferation of atypical, poorly glandular epithelium. The enzyme antibody method showed that both chromogranin A and GATA3 were positive, indicating parathyroid tissue. Only a few Ki-67-positive cells were noted. The maximum diameter of the specimen was about 1.2 cm with a regular margin. There were no signs of mitosis or fibrous septum, suggesting a low possibility of malignancy (Figures 2A-2D). Based on the lack of swelling of other parathyroid glands in the cervical area, we considered that the above pathological findings were consistent with the final diagnosis of ectopic parathyroid adenoma.

The serum calcium and PTH levels remained in their normal ranges thereafter, and the patient gave birth at 38 weeks and one day. A female infant was born weighing 2472 g, with a length of 46.5 cm, umbilical arterial blood gas pH of 7.272, and an Apgar score of 8/9. After delivery, neither the mother nor infant required calcium supplementation. While the infant had midgut malrotation, and so underwent surgical correction shortly after birth, both the mother and infant followed a favorable course, being free from any endocrinological disorders.

In this case, although hypercalcemia is widely accepted as a cause for gastrointestinal symptoms, the notable aspect of this case is the persistence of these symptoms even after successful correction of the hypercalcemia [12,13]. To elucidate unknown factors responsible for the persistent symptom due to the parathyroid adenoma, we performed serial measurements of potential factors contributing to gastrointestinal motility.

**Figure 3:**
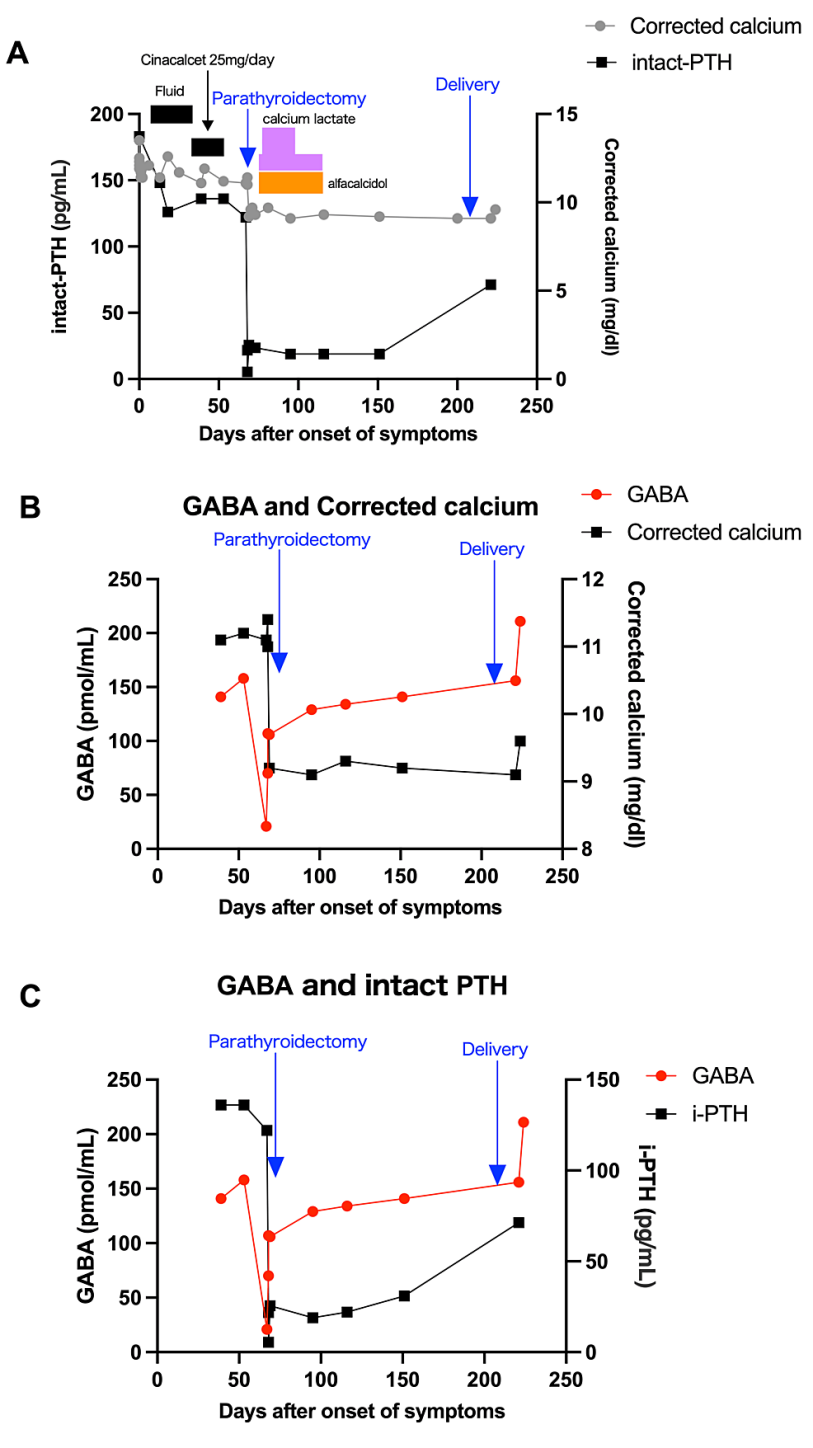
Dynamic monitoring of serum calcium, intact PTH, and GABA levels during treatment and critical care periods. (A) Trends in serum calcium and intact PTH in the overall treatment course, (B) serial trends of serum GABA and corrected calcium, and, (C) serial trends of serum GABA and intact PTH in perioperative and perinatal periods. PTH: parathyroid hormone; GABA: gamma-aminobutyric acid

Several recent emerging studies suggested that GABA is co-localized and co-produced with PTH in parathyroid gland cells [14,15]. Based on reports that GABA and its receptors regulate gastrointestinal motility via enteric GABAergic neurons, we conducted serial measurements of serum GABA from the preoperative to post-partum periods using conserved frozen serum samples [16-18]. Serum GABA levels were measured using the high-performance liquid chromatography (HPLC) method (Tokyo, Japan: SRL, Inc.). For comparison, we also analyzed the levels of gastrin and calcitonin in the same samples [19,20]. GABA shows a synchronized trend with calcium at most points and with intact PTH (Figures 3A-3C), while gastrin and calcitonin showed different trends from calcium and PTH (data not shown).

## Discussion

Here, we described the case of a patient with gestational ectopic PHPT who presented with gastrointestinal symptoms in the first trimester of pregnancy. We conducted serial measurements of GABA perioperatively and perinatally. In this case, the rarity of the coexistence of ectopic parathyroid adenoma plus PHPT in pregnancy necessitated careful decision-making regarding the treatment plan to minimize any harmful effects on pregnancy.

A recent case series of PHPT in pregnancy reported that over half of the cases in this series were diagnosed incidentally, and the overlap with typical pregnancy symptoms, such as hyperemesis gravidarum means that it tends to go unnoticed [21]. The origin of the parathyroid glands is the endoderm, which develops from the dorsal wing of the third and fourth pharyngeal pouch [22].

Ectopic PHPT is reportedly present in 6-16% of hyperparathyroid adenoma cases, and the anterior mediastinum is one of the most common sites [23]. In the present case, the absence of parathyroid adenoma prompted us to conduct a systemic study to identify the site of the adenoma, which is essential for surgery planning.

While there are multiple points to be discussed regarding the present case, we consider that the following needed to be considered comprehensively: surgical indication for gestational PHPT, controlling calcium levels during pregnancy, and the significance of GABA level measurement.

First, regarding the desirable timing of surgery for a patient with gestational PHPT, the second trimester is considered to be optimal [24]. While medical therapy can be initiated in symptomatic pregnant PHPT patients, the failure of treatment can be an indication for surgery regardless of the gestational age of 20 years. In this case, a limited decline of calcium after fluid administration and medication with cinacalcet prompted us to conduct surgical intervention.

Second, we decided on surgical resection of the parathyroid adenoma even though the serum calcium level was 11-12 mg/dL after the initial fluid administration. This was based on previous studies reporting a 3.5-times increase in miscarriage in pregnant females with PHPT, especially with a calcium level exceeding 11.4 mg/dL [11]. Another recent pilot study reported a 0.34% prevalence of undiagnosed PHPT in pregnant females with recurrent miscarriages (≥3 consecutive miscarriages under 24 weeks of gestation) [25]. These studies suggest the necessity of therapeutic intervention for pregnant females with hypercalcemia and PHPT.

Third, to our knowledge, this is the first report measuring GABA perioperatively and perinatally in a case of gestational PHPT. As described above, in a recent study, GABA was considered to be synthesized from glutamate by GAD1/2 in parathyroid gland cells to activate GABAB1R in GABAB1R/CaSR heteromers to regulate PTH secretion in an autocrine manner [14]. Although the role of GABA in hyperparathyroidism largely remains to be elucidated, histological studies of 65 PHPT patients who underwent parathyroidectomy demonstrated significantly decreased expressions of GABABR1 and CaSR in PHPT patients with parathyroid adenomas compared with healthy controls [13].

On the other hand, limited data are available regarding the significance of GABA levels during pregnancy. Although studies involving animal models reported the potential effects of GABA administration on fetal development, how the dynamics of GABA levels in the peripartum period affect the mother and infant regarding their endocrinological outcomes remains poorly understood [26,27]. Careful evaluation is necessary to interpret our results.

In summary, we report the case of a patient with gestational ectopic PHPT who underwent surgical resection of parathyroid adenoma in the second trimester. A collection of cases is warranted to clarify the relevance of GABA and the utility of its measurement for the control of gastrointestinal symptoms in gestational PHPT.

## Conclusions

In this study, we have illustrated a complex instance of gestational hypercalcemia resulting from PHPT, successfully managed through timely surgical intervention. The uniqueness of this case lies in its demonstration of ectopic PHPT during pregnancy, a condition that requires astute clinical acumen for early detection and effective management to ensure favorable maternal and fetal outcomes.

The present study also emphasizes the importance of a multidisciplinary approach in the diagnosis and management of PHPT during pregnancy, highlighting the potential benefits of exploring novel biomarkers such as GABA in managing complex cases. Continued research and case accumulation are essential to deepen our understanding of PHPT in pregnancy and to refine the strategies for its management, ultimately improving care for affected patients and their offspring.

## References

[1] Minisola S, Arnold A, Belaya Z (2022). Epidemiology, pathophysiology, and genetics of primary hyperparathyroidism. J Bone Miner Res.

[2] Walker MD, Silverberg SJ (2018). Primary hyperparathyroidism. Nat Rev Endocrinol.

[3] Yeh MW, Ituarte PH, Zhou HC (2013). Incidence and prevalence of primary hyperparathyroidism in a racially mixed population. J Clin Endocrinol Metab.

[4] Newey PJ, Nesbit MA, Rimmer AJ (2012). Whole-exome sequencing studies of nonhereditary (sporadic) parathyroid adenomas. J Clin Endocrinol Metab.

[5] Costa-Guda J, Arnold A (2014). Genetic and epigenetic changes in sporadic endocrine tumors: parathyroid tumors. Mol Cell Endocrinol.

[6] Brandi ML, Agarwal SK, Perrier ND, Lines KE, Valk GD, Thakker RV (2021). Multiple endocrine neoplasia type 1: latest insights. Endocr Rev.

[7] Malekar-Raikar S, Sinnott BP (2011). Primary hyperparathyroidism in pregnancy - a rare cause of life-threatening hypercalcemia: case report and literature review. Case Rep Endocrinol.

[8] Bollerslev J, Rejnmark L, Zahn A (2022). European expert consensus on practical management of specific aspects of parathyroid disorders in adults and in pregnancy: recommendations of the ESE educational program of parathyroid disorders. Eur J Endocrinol.

[9] Horton WB, Stumpf MM, Coppock JD (2017). Gestational primary hyperparathyroidism due to ectopic parathyroid adenoma: case report and literature review. J Endocr Soc.

[10] Sharma SG, Levine SN, Yatavelli RK, Shaha MA, Nathan CA (2020). Parathyroidectomy in first trimester of pregnancy. J Endocr Soc.

[11] Norman J, Politz D, Politz L (2009). Hyperparathyroidism during pregnancy and the effect of rising calcium on pregnancy loss: a call for earlier intervention. Clin Endocrinol (Oxf).

[12] Islam AK (2021). Advances in the diagnosis and the management of primary hyperparathyroidism. Ther Adv Chronic Dis.

[13] Almuradova E, Cicin I (2023). Cancer-related hypercalcemia and potential treatments. Front Endocrinol (Lausanne).

[14] Hong AR, Kim YA, Bae JH (2016). A possible link between parathyroid hormone secretion and local regulation of GABA in human parathyroid adenomas. J Clin Endocrinol Metab.

[15] Chang W, Tu CL, Jean-Alphonse FG (2020). PTH hypersecretion triggered by a GABA(B1) and Ca(2+)-sensing receptor heterocomplex in hyperparathyroidism. Nat Metab.

[16] Kawakami S, Uezono Y, Makimoto N, Enjoji A, Kaibara M, Kanematsu T, Taniyama K (2004). Characterization of GABA(B) receptors involved in inhibition of motility associated with acetylcholine release in the dog small intestine: possible existence of a heterodimer of GABA(B1) and GABA(B2) subunits. J Pharmacol Sci.

[17] Krantis A (2000). GABA in the mammalian enteric nervous system. News Physiol Sci.

[18] Auteri M, Zizzo MG, Serio R (2015). GABA and GABA receptors in the gastrointestinal tract: from motility to inflammation. Pharmacol Res.

[19] Jaffe BM, Peskin GW, Kaplan EL (1974). Relationship of serum gastrin to parathyroid hormone secretion in sheep. Metabolism.

[20] Bolman RM 3rd, Cooper CW, Garner SC, Munson PL, Wells SA Jr (1977). Stimulation of gastrin secretion in the pig by parathyroid hormone and its inhibition by thyrocalcitonin. Endocrinology.

[21] DiMarco AN, Meeran K, Christakis I, Sodhi V, Nelson-Piercy C, Tolley NS, Palazzo FF (2019). Seventeen cases of primary hyperparathyroidism in pregnancy: a call for management guidelines. J Endocr Soc.

[22] Mohebati A, Shaha AR (2012). Anatomy of thyroid and parathyroid glands and neurovascular relations. Clin Anat.

[23] Shao Y, Zeng Q, Lv B, Chen X, Sheng L (2022). Case report: primary hyperparathyroidism due to posterior mediastinal parathyroid adenoma with one-year follow-up. Front Surg.

[24] Saad AF, Pacheco LD, Costantine MM (2014). Management of ectopic parathyroid adenoma in pregnancy. Obstet Gynecol.

[25] DiMarco A, Christakis I, Constantinides V, Regan L, Palazzo FF (2018). Undiagnosed primary hyperparathyroidism and recurrent miscarriage: the first prospective pilot study. World J Surg.

[26] Tian N, Liang H, Luo W (2020). GABA consumption during early pregnancy impairs endometrial receptivity and embryo development in mice. J Biochem Mol Toxicol.

[27] Chen W, Zhang Q, Wang H, Tan D, Tan Y (2021). Unique and independent role of the GABA(B1) subunit in embryo implantation and uterine decidualization in mice. Genes Dis.

